# Psychosocial Impact of SARS

**DOI:** 10.3201/eid1007.040090

**Published:** 2004-07

**Authors:** Hector W.H. Tsang, Rhonda J. Scudds, Ellen Y.L. Chan

**Affiliations:** * The Hong Kong Polytechnic University, Hong Kong

**Keywords:** SARS, psychosocial impact, family burden, letter

**To the Editor:** An outbreak of severe acute respiratory syndrome (SARS) occurred from February to May 2003 in Hong Kong, China, Singapore, and Canada. According to the World Health Organization, 1,755 people were infected in Hong Kong; 386 of these were healthcare workers. A total of 300 persons died from SARS, constituting a death rate of 17% (1).

Evidence suggests that persons infected with SARS recovered physically, but SARS is associated with social and psychological problems poorly understood by the scientific community. A survey in a convalescent hospital in Hong Kong showed that approximately 50% of recovered SARS patients showed anxiety (2), and approximately 20% were fearful (2). Approximately 20% of the rehabilitated patients showed some negative psychological effects (3), which included insomnia and depression. Some patients with serious cases could not rid themselves of the memories of fighting SARS, and these memories disrupted their daily activities. These psychosocial problems may be due to the complications of SARS medications, such as ribavirin and corticosteroid. Persons who took these drugs had hair loss, major memory loss, impaired concentration, and depression. A medical practitioner in Hong Kong who recovered from SARS attempted suicide because complications from drugs made him unable to earn his living (4).

In addition to SARS patients themselves, an estimated 50% of family members of SARS patients had psychological problems, including feelings of depression or stigmatization (5). They had difficulties sleeping, and some children who had lost parents cried continuously. Some children also felt embarrassed to be a member of a SARS family (6). The spouse of one healthcare worker who died from SARS attempted suicide at her workplace (7). The loss of parents who were SARS patients also impaired the growth of their children (7). A study conducted in China (8) reported that negative SARS-related information increased persons' perception of their risk and led to irrational nervousness or fear.

Although data from systematic studies of SARS do not exist, evidence suggests that this disease has psychosocial consequences for SARS patients, their families, and society. While biomedical scientists must continue their efforts to clarify the genetic makeup of the SARS coronavirus, look for new medications, and develop vaccines (9–13), the social and psychological aspects of SARS should not be overlooked. Since nearly all resources are devoted to biomedical research and medical treatment, psychosocial problems of SARS patients and their families are largely ignored. Our review of the literature using the ISI Web of Knowledge on January 17, 2004, substantiated this observation. To date, no systematic study examining psychosocial consequences of SARS has been published in scientific journals. A systematic exploration of how SARS negatively affects patients' mental health is needed so that appropriate interventions may be implemented at individual, family, and societal levels.
